# International tuberculosis contact-tracing notifications in Germany: analysis of national data from 2010 to 2018 and implications for efficiency

**DOI:** 10.1186/s12879-020-04982-z

**Published:** 2020-04-06

**Authors:** Saskia Glasauer, Stefan Kröger, Walter Haas, Nita Perumal

**Affiliations:** 1 Institute for Medical Information Processing, Biometry and Epidemiology, Ludwig-Maximilians-Universität Munich, Munich, Germany; 2 Department of Infectious Disease Epidemiology, Robert Koch Institute, Berlin, Germany

**Keywords:** Tuberculosis, Contact-tracing, Public health

## Abstract

**Background:**

International contact-tracing (CT) following exposure during long-distance air travel is resource-intensive, whereas evidence for risk of tuberculosis (TB) transmission during international travel is weak. In this study, we systematically analyzed the information from international requests for CT received at the national level in Germany in order to evaluate the continued utility of the current approach and to identify areas for improvement.

**Methods:**

An anonymized archive of international CT notifications received by the Robert Koch Institute between 2010 and 2018 was searched for key parameters for data collection. A total of 31 parameters, such as characteristics of TB patients and their identified contacts, were extracted from each CT notification and collated into a dataset. Descriptive data analysis and trend analyses were performed to identify key characteristics of CT notifications, patients, and contacts over the years.

**Results:**

192 CT notifications, each corresponding to a single TB index case, were included in the study, increasing from 12 in 2010 to 41 in 2018. The majority of notifications (*N* = 130, 67.7%) concerned international air travel, followed by private contact (*N* = 39, 20.3%) and work exposure (*N* = 16, 8.3%). 159 (82.8%) patients had sputum smear results available, of which 147 (92.5%) were positive. Of 119 (62.0%) patients with drug susceptibility testing results, most (*N* = 92, 77.3%) had pan-sensitive TB, followed by 15 (12.6%) with multi-drug resistant TB. 115 (59.9%) patients had information on infectiousness, of whom 99 (86.1%) were considered infectious during the exposure period. 7 (5.3%) patients travelled on long-distance flights despite a prior diagnosis of active TB. Of the 771 contact persons, 34 (4.4%) could not be reached for CT measures due to lack of contact information.

**Conclusion:**

The high variability in completeness of information contained within the international CT requests emphasizes the need for international standards for reporting of CT information. With the large proportion of TB patients reported to have travelled while being infectious in our study, we feel that raising awareness among patients and health professionals to detect TB early and prevent international long-distance travel during the infectious disease phase should be a cornerstone strategy to safeguard against possible transmission during international travel.

## Background

Epidemiological contact-tracing (CT) is considered to be crucial for the prevention of further transmission of a number of infectious diseases [1]. CT is defined as the identification and examination of relevant contacts of infectious cases, testing for the presence of infection or disease and, if necessary, provision of appropriate therapy before the occurrence of serious illness [2]. With accessibility to international travel increasing worldwide – in 2017, 4.1 billion passengers flew on international flights – the risk of spread of communicable diseases by infectious travelers has increased as well [3, 4]. With 1.2 million deaths in 2018, TB is the leading cause of death from a single infectious agent worldwide [5]. In 2018, 7.0 million people were newly diagnosed with TB and it is estimated that about one quarter of the world’s population is latently infected with TB [5]. TB is transmitted from person to person via small droplets in the air (‘aerosols’ < 5 μm in diameter) and the risk of infection depends on the infectiousness of the TB case, the duration and intensity of contact, and the susceptibility of the contact person [2, 6]. Germany is considered a low-incidence country for TB, with 5486 notified TB cases (incidence of 6.7 per 100,000) notified in 2017 to the national public health institute, the Robert Koch Institute (RKI) [7]. Several studies have shown that also in low incidence countries, new cases of TB can be attributed to recent transmission [8–13]. For example, recent transmission accounts for about 13% of TB cases in the US, about 23% of TB cases in Belgium, and up to one third of all new TB cases in London [8, 9, 13]. As a result, early detection of new TB cases and the prevention of further transmission of TB is a crucial component in many TB control plans in low incidence countries [2, 3, 14].

As TB, and especially drug resistant TB, has the potential to pose a serious public health risk, the International Health Regulations (IHR), under Article 44, provide the legal framework for public health authorities to exchange information regarding patients and exposed contacts in situations where cases of active TB are known to have travelled internationally without taking appropriate infection protection measures [15, 16]. While the IHR primarily focus on infectious diseases during air travel, Article 44 also provides the basis for international exchange on non-flight related events, such as private contact or contact during ground transport or sea travel [15].

**Fig. 1:**
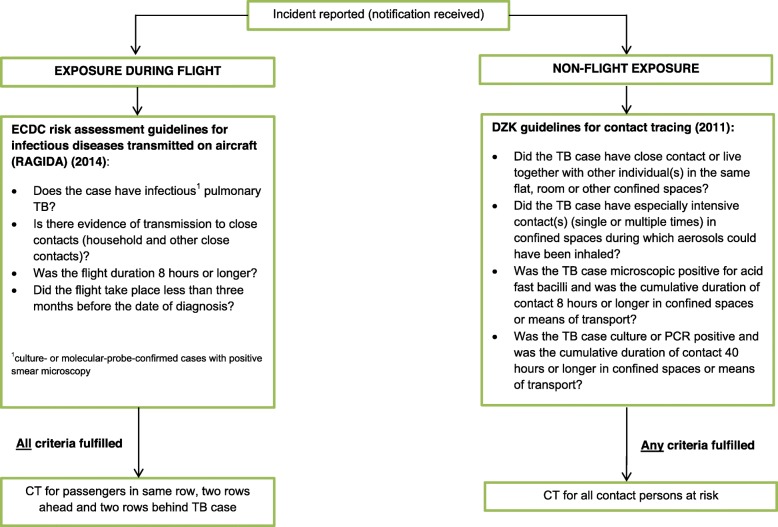
Risk assessment algorithm applied at the Robert Koch Institute for initiation of international tuberculosis contact-tracing

When it comes to the current available evidence on this topic, most of the literature focuses on CT following air travel and several systematic reviews and research articles have aimed to quantify the risk of transmission [6, 8, 17–26]. A systematic review by Kotila and colleagues reported a very low risk estimate of 0.1–1.3% for testing positive for TB infection following exposure on a long-distance flight of > 8 h in duration [18] and so far no case of active TB disease following transmission on aircraft has been reported [6, 17, 18]. Nonetheless, international CTs involving air-travel are prioritized and conducted regularly in a number of countries and the European Center for Disease Prevention and Control (ECDC) developed a risk assessment guidance for infectious diseases transmitted on aircraft (RAGIDA) in 2009 (latest update in 2014), including a version specifically for TB CT on aircraft [27]. Literature investigating TB transmission during international travel settings other than air-travel, such as using ground travel or during group tours, is rather limited and, similar to air-travel, high-quality evidence is lacking. In 2011, the German Central Committee against Tuberculosis (DZK) published new recommendations for TB CT in Germany, including criteria for the assessment of initiation of CT [2], which can also be applied to international non-flight CT conditions. As a result, RKI has established a risk assessment algorithm based on the criteria set-out in these documents to assess the need for CT in Germany following notifications from other countries regarding international TB cases (Fig. 1).

Given the weak evidence so far for the risk of TB transmission during international travel and the limited public health resources available for TB prevention [6], reevaluation of the strategy of international contact tracing for TB is urgently needed. In Germany, CT notification data has so far not been systematically analyzed; hence, this study aims to collate and analyze international contact tracing notifications received by Germany from other countries in order to facilitate priority-setting for CT, with a special focus on air travel. The objectives of this study are i) to create an inventory of the type and quantity of international CT notifications received by the RKI, ii) to summarize the characteristics of the TB cases and their contacts in Germany, and iii) to assess the quality and completeness of the CT information received in order to evaluate the continued utility of the current approach and to identify areas for improvement.

## Methods

### Data background

In Germany, CT is the responsibility of the local health authorities. RKI, as the national public health institute, is often, but not always, the contact point between international health authorities and the local health authorities for cases where individuals with German citizenship or those residing in Germany are identified as part of an international CT for TB. Foreign health authorities usually send the information regarding the event (i.e. an international CT notification) to the RKI via the secure Early Warning and Response System (EWRS) of the European Union or by email, with any personal information being encrypted or password-protected. As there is no international standard on the reporting of CT information for TB, there are large differences in the extent and detail of the information contained within these notifications. However, they usually contain information on the type of exposure, clinical and demographic characteristics of the TB patient (i.e. the index case) and contact information of the identified contact persons. Based on the provided information on the contact person(s), i.e. name, date of birth, address, telephone number or passport number, RKI then identifies the responsible German local health authority for each contact person and forwards the CT notification to them for further investigation and follow-up. Before 2018, RKI forwarded every international CT notification to the responsible local health authorities, as it was considered the responsibility of the notifying foreign health authority to assess whether CT should be initiated. However, since not all countries apply the same risk assessment algorithm as RKI for international CT for TB, RKI undertook the decision in 2018 to double-check each international CT notification against the risk assessment algorithm described in Fig. 1 in order to assess whether a CT should be a conducted, before forwarding the notifications that met the criteria to the local health authorities. In case of incomplete data for criteria assessment, the foreign health authority is asked to provide this information; if the information remains missing, the notification is deemed to have not met the algorithm criteria and CT is not conducted, unless there are exceptional circumstances, e.g. a severely ill index case or new relevant information becomes available at a later time point.

### Data source and collection

An anonymized archive containing key parameters/metadata of all CTs notified to the RKI since 2010 was utilized in this study. First, the archive was inspected and important parameters for systematic extraction were identified (Table 1). Finally, each CT notification within the archive was searched for the presence of the included parameters/variables and corresponding information was extracted (Table 1).

**Table 1:** List of key parameters extracted from international tuberculosis contact-tracing notifications received by RKI, 2010–2018

**CT notification**	• Notifying country
	• Month of notification
	• Year of notification
**Index case**	General characteristics:
	• Age
	• Sex
	• Nationality
	Clinical characteristics:
	• TB diagnosis (type of TB, site of disease)
	• Smear microscopy (positive, negative)
	• Culture (positive, negative, pending)
	• PCR (positive, negative, pending)
	• Chest X-ray (positive, negative, cavitary lesions)
	• Presence of drug susceptibility testing result (yes/no)
	• Drug resistance profile (H^a^-resistant), (R^a^-resistant), (S^a^-resistant), MDR-TB^b^, XDR-TB^c^
	• Infectiousness during contact (yes/no)
	• Information on symptoms during contact (yes/no)
	• Information on transmission to close contacts (yes/no)
**Exposure**	• Responsible local health authority in Germany
	• Federal state of responsible local health authority
	• Type of contact (flight contact, private contact, work contact, school/education-related contact, ship contact)
	• Time between exposure and diagnosis (< 3 months, > 3 months)
	• Duration of contact (< 8 h, > 8 h, < 40 h, > 40 h)
	• Departure city^d^
	• Departure country^d^
	• Arrival city^d^
	• Arrival country^d^
	• Contact person seated within two rows of index case (yes/no)^d^
**Contact person(s)**	• Information on name (yes/no)
	• Information on passport number (yes/no)
	• Information on address (yes/no)
	• Information on telephone number (yes/no)
	• Information on email address (yes/no)
	• Child (yes/no)

^a^ H: Isoniazid, R: Rifampicin, S: Streptomycin

^b^ Multi-drug resistant tuberculosis (MDR-TB) is defined as resistance to at least Isoniazid and Rifampicin

^c^ Extensively drug resistant tuberculosis (XDR-TB) is defined as MDR-TB plus resistance against one fluoroquinolone and one injectable

^d^ Information only collected for air travel

### Case inclusion and variable definitions

To be included in the analysis, CT notifications must have originated from a foreign health authority and received by the RKI between 2010 and 2018. TB patients that were described in these notifications were defined as index cases.

A long-distance flight was defined as flight duration > 8 h. Countries were categorized as high or low TB burden countries in accordance with the categorization of the World Health Organization, as published by the Stop TB Partnership in 2019 [28]. An index case was considered as having been infectious during contact when he/she was classified as such by the responsible foreign health authority, i.e. reported as being “infectious” in the CT notification.

### Data analysis

The collated CT information was examined using descriptive data analysis. Completeness of information was analyzed and reported as case numbers and proportions. For categorical variables, tables were derived summarizing case numbers and proportions of the different categories. Proportions were calculated in relation to the number of cases with available information for their respective category (denominator). Continuous variables were examined by calculating medians and corresponding ranges.

To analyze if there was a difference in infectiousness between index cases from countries with high versus low burden of TB, Fisher’s exact test was used. Linear trends from 2010 to 2018 in the proportion of index cases diagnosed with TB and MDR-TB and in the proportion of flight versus non-flight exposure were analyzed using the Chi^2^-test for trends. To study whether there was a monotonic trend in the median number of contact persons per index case between 2010 and 2018, the non-parametric Mann-Kendall test was used. For all tests, a *p*-value of < 0.05 was considered significant. All analyses were conducted using RStudio version 3.5.1.

All data were collected in accordance with the International Health Regulations (IHR 2005), Article 44 on “Collaboration and assistance” and data protection guidelines were strictly followed. Informed consent was deemed not necessary as data were fully anonymized before analysis.

## Results

**Fig. 2:**
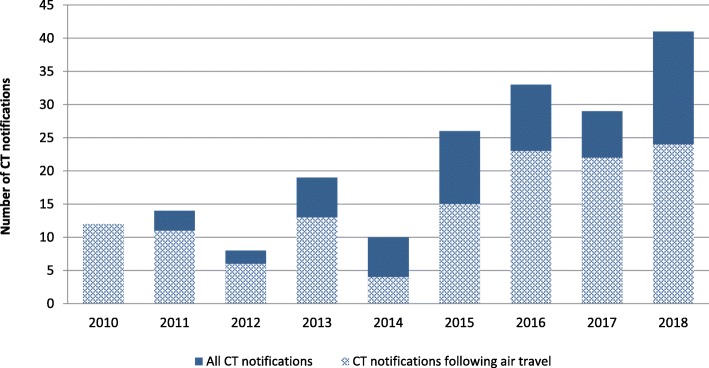
Number of international tuberculosis contact-tracing notifications received, 2010–2018 (*N* = 192), with share of air-travel related notifications

Between 2010 and 2018, RKI received 209 international notifications regarding TB patients from 23 different countries. Of these, 17 notifications were excluded from the analyses as they concerned TB patients moving to Germany from another country (*N* = 15), 1 German resident being diagnosed abroad with TB and 1 German resident being diagnosed abroad and being repatriated back to Germany due to TB. Of the remaining 192 notifications, most were received from the United States (*N* = 38), followed by Singapore (*N* = 24), Canada (*N* = 18), France (*N* = 14) and Austria (*N* = 13). Figure 2 displays the total number of international CT notifications per year received by the RKI, with CT notifications exclusively regarding air travel shown as share of all notifications. At the start of the study period in 2010, RKI received 12 international CT notifications from foreign health authorities. These increased to 26 in 2015 and, in 2018, at the end of the study period, RKI received 41 CT notifications in total, which is more than double the number in 2010. Of the overall 192 notifications, the majority (*N* = 130, 67.7%) concerned international air travel, followed by 39 (20.3%) international CT notifications related to private contact, such as family/friend visits abroad. Sixteen (8.3%) notifications concerned work-related exposure, 6 (3.1%) involved school/education related exposure and 1 (0.5%) concerned exposure during a sea cruise. No notifications regarding exposure during ground travel were received by the RKI during the study period. There was no significant linear trend in the proportion of notifications concerning air travel; similarly, there was no significant linear trend in the proportion of notifications concerning non-flight exposure, i.e. personal, school/education related, and work contact (Chi^2^-test for trend *p* > 0.05).

Table 2 shows the completeness of information of demographic and clinical characteristics of TB index cases and their contacts from 2010 to 2018 when applying the CT risk assessment algorithm at RKI. Of the 130 index cases who travelled on international long-distance flights, only 15 (11.5%) had information on all RAGIDA criteria for the assessment of CT following air travel. Of the 62 index cases with other exposures, for which the DZK CT recommendations were applicable, only 10 (16.1%) notifications provided information on cumulative duration of exposure and either smear microscopy results or laboratory culture results for the index case, which are important to assess the need for CT for incidents that are not related to air travel.

**Table 2:** Completeness of information on demographic and clinical characteristics of TB index cases and their contacts

Characteristics	Information available	
	N	%
**Index cases – demographic characteristics (N = 192)**		
Sex	98	51.0%
Age	75	39.1%
Nationality	48	25.0%
**Index cases – lab and clinical characteristics (N = 192)**		
Smear microscopy	159	82.8%
Chest X-ray	109	56.8%
Diagnosis	119	62.0%
Time between exposure and diagnosis	133	69.3%
Infectiousness during exposure	115	59.9%
Symptoms during exposure	81	42.2%
**Index cases – air travel (N = 130)**		
Smear microscopy	112	86.2%
Flight duration	125	96.2%
Time between flight and diagnosis	107	82.3%
Transmission to close contacts	18	13.8%
All RAGIDA criteria	15	11.5%
**Index cases – other exposure (*N*** **= 62)**		
Smear microscopy	49	79.0%
Culture	25	40.3%
Duration of exposure	19	30.6%
Time between exposure and diagnosis	25	40.3%
Duration of exposure and either microscopy results or culture results	10	16.1%
**Availability of Information on identified contacts (*N*** **= 771)**		
Any contact information	742	96.2%
Sufficient contact information for CT	695	90.1%

Table 3 provides a summary of the characteristics of TB index cases and their identified contacts, as described in the international TB CT notifications received by RKI, 2010–2018. The 192 notifications corresponded to 192 index cases. Ninety-eight (51.0%) index cases had information on sex available, of which 56 (57.1%) were male. Age was reported for 75 (39.1%) index patients, with a median age of 31 years (range: 16–79 years). Information on nationality was available in 48 (25.0%) notifications, accounting for 29 different nationalities. Four (8.3%) index patients were from the Americas, 6 (12.5%) were from Africa, another 6 (12.5%) were from Asia, 8 (16.7%) were from Eastern Europe, and 24 (50.0%) were from Western Europe.

**Table 3:** Characteristics of TB index cases and their identified contacts, 2010–2018

		2010	2011	2012	2013	2014	2015	2016	2017	2018	Total
Total number of index cases		12	14	8	19	10	26	33	29	41	192
**Index cases – demographic characteristics**											
Sex (*N* = 98)	Male	2	4	1	6	4	7	11	13	8	56 (57.1%)
	Female	8	4	1	1	3	6	9	4	6	42 (43.9%)
	Unknown	2	6	6	12	3	13	13	12	27	94
Median age		23.0	31.5	41.5	35.5	31.0	29.0	31.5	31.0	55.0	31.0
Nationality (region) (*N* = 48)	Africa	0	0	0	1	1	2	0	1	1	6 (12.5%)
	The Americas	2	1	0	0	1	0	0	0	0	4 (8.3%)
	Asia	0	0	0	0	0	2	2	1	1	6 (12.5%)
	Eastern Europe	0	0	0	0	0	2	2	2	2	8 (16.7%)
	Western Europe	0	1	3	1	2	5	4	3	5	24 (50.0%)
	Unknown	10	12	5	17	6	15	25	22	32	144
**Index cases – clinical characteristics**											
Smear microscopy (*N* = 159)	Positive	12	12	6	15	10	19	25	21	27	147 (92.5%)
	negative	0	1	1	4	0	2	1	2	1	12 (7.5%)
	Unknown	0	1	1	0	0	5	7	6	13	33
Culture (*N* = 102)	Positive	8	9	3	13	4	6	17	9	10	79 (77.5%)
	Negative	0	0	0	0	0	0	0	0	0	0 (0.0%)
	Pending	0	1	3	1	2	3	3	5	5	23 (22.5%)
	Unknown	4	4	2	5	4	17	13	15	26	90
Chest X-ray (*N* = 109)	Normal	0	0	1	0	0	3	0	1	0	5 (4.6%)
	Abnormal	2	5	2	7	3	4	7	6	5	41 (37.6%)
	Cavitary lesions	7	3	3	9	4	4	16	7	10	63 (57.8%)
	Unknown	3	6	2	2	4	15	10	15	26	83
Diagnosis (*N* = 119)	Pan-sensitive TB	7	10	4	10	5	11	14	12	19	92 (77.3%)
	Mono-resistant TB	1	1	0	3	0	3	1	1	1	11 (9.2%)
	MDR-TB	1	0	0	1	1	2	7	3	0	15 (12.6%)
	XDR-TB	0	0	0	0	0	0	1	0	0	1 (0.8%)
	Unknown	3	3	4	5	4	10	10	13	21	73
Infectious during exposure (*N* = 115)	Yes	10	6	6	11	4	12	18	14	18	99 (86.1%)
	No	1	2	1	2	0	2	1	5	2	16 (13.9%)
	Unknown	1	6	1	6	6	12	14	10	21	77
Symptoms during exposure (*N* = 81)	Yes	9	3	3	4	2	6	11	11	13	62 (76.5%)
	No	1	1	0	2	1	3	2	5	4	19 (23.5%)
	Unknown	2	10	5	13	7	17	20	13	24	111
**Exposure Setting**											
Type of exposure (N = 192)	Flight contact	12	11	6	13	4	15	23	22	24	130 (67.7%)
	Private contact	0	2	2	3	4	4	4	5	15	39 (20.3%)
	Work contact	0	1	0	2	1	6	2	2	2	16 (8.3%)
	School- or education-related contact	0	0	0	1	1	1	3	0	0	6 (3.1%)
	Ship contact	0	0	0	0	0	0	1	0	0	1 (0.5%)
**Contact persons**											
Total number of contact persons		30	88	52	86	48	80	104	186	97	771
Child contacts		0 (0.0%)	3 (3.4%)	7 (13.5%)	0 (0.0%)	1 (2.1%)	2 (2.5%)	6 (5.8%)	5 (2.7%)	8 (8.2%)	32 (4.2%)
Number of contact persons who could not be traced		3 (0.1%)	7 (8.0%)	1 (1.9%)	1 (1.2%)	20 (4.2%)	0 (0.0%)	0 (0.0%)	0 (0.0%)	2 (2.1%)	34 (4.4%)

Sputum smear results were reported for 159 (82.8%) of the 192 index cases, of which 147 (92.5%) were sputum smear positive. Chest radiograph results were reported for 109 (56.8%) index cases, with 63 (57.8%) having cavitary disease. Drug susceptibility testing (DST) results were reported for 119 (62.0%) index cases. The majority of index cases (*N* = 92, 77.3%) had pan-sensitive TB, followed by 15 (12.6%) multi-drug resistant (MDR-TB) cases, 6 (5.0%) cases resistant to Isoniazid, 4 (3.4%) cases resistant to Rifampicin, 1 (0.8%) case resistant to Streptomycin and 1 (0.8%) extensively drug-resistant (XDR-TB) case. Neither the proportion of pan-sensitive TB nor the proportion of MDR-TB diagnoses among all index cases showed a significant trend over time (Chi^2^-test for trend *p* > 0.05). Foreign health authorities reported information on infectiousness during exposure for 115 (59.9%) index cases, of which 86.1% (*N* = 99) were classified as having been infectious during exposure. There was no significant difference in the number of index cases who were reported as being infectious between those with nationalities from high TB-burden countries compared to those with nationalities from low burden countries (Fisher’s exact test *p* > 0.05). Information on time between exposure and diagnosis was available for 133 (69.3%) index cases, the majority (*N* = 114, 85.7%) of whom were diagnosed within 3 months of the contact reported in the CT notification. Seven (5.3%) index cases, two of whom had MDR-TB and all of whom flew on international long-distance flights, were diagnosed with TB prior to the reported contact (i.e. flight exposure). Since the stricter application of the RKI risk assessment algorithm, 7 of the 41 (17.1%) international CT notifications in 2018 were deemed to not meet the CT criteria: 5 due to too long time between exposure and diagnosis and 2 due to missing information. Therefore, these notifications were not forwarded to local health authorities for further investigation.

Overall, 771 contacts were identified for the 192 index cases; the median number of contact persons was 2 per index case (range: 1–87). There was no significant trend in the median number of contact persons between 2010 and 2018 (Mann-Kendall test *p* > 0.05). The 771 contacts fell under the jurisdiction of 180 different local health authorities in Germany, with the local health authorities in Berlin (*N* = 68), Munich (*N* = 40), Hamburg (*N* = 39), Frankfurt (*N* = 27), and Stuttgart (*N* = 16) being responsible for the most contact persons, largely reflecting the large population sizes of these cities and their major international airports. Of these, 514 (66.7%) contact persons were exposed to a TB index patient during a long-distance flight, 160 (20.8%) had private contact with an index case, 77 (10.0%) had a work-related contact, 19 (2.5%) were contacts during a school/education-related event, and 1 (0.01%) was in contact with an index patient on a cruise ship. For 695 (90.1%) contacts, CTs could be initiated based on the information provided by the foreign health authorities, such as passport number, address or other contact details (e. g. telephone number or email address). Thirty-four (4.4%) contact persons could not be investigated due to insufficient data for tracing, with 29 (3.8%) contacts having no contact information at all.

Of the 771 contacts, 32 (4.2%) were children. Eighteen children (56.3%) had private contact with an index case, 10 (31.3%) were exposed during a long-distance flight, and 4 (12.5%) were exposed during contact with their nannies. Four (12.5%) children had contact with a drug-resistant index case, of which 1 was an MDR-TB case, 2 Rifampicin-resistant cases, and 1 Streptomycin-resistant case. Five (15.6%) children could not be investigated due to lack of contact or address information, 3 of whom were exposed to drug-resistant TB cases (2 Rifampicin-resistant and 1 Streptomycin-resistant).

## Discussion

Our study is the first to systematically collate and analyze data regarding international CT notifications for TB at a nationwide level in Germany and is, to the best of our knowledge, the first of its kind for TB worldwide. Between 2010 and 2018, RKI received notifications from 23 different countries, involving 192 TB index cases and 771 corresponding contact persons. International CT notifications steadily increased in number over the study period, from 12 in 2010 to 41 in 2018; this likely corresponded to an increase in global air traffic, but perhaps also increased awareness and communication regarding TB. The majority of the notifications (*N* = 130, 67.7%) concerned exposure to an index case with active TB during long-distance air travel, accounting for 514 (60.0%) of identified contacts during the study period. However, compared to an overall LTBI prevalence of 28% among investigated contacts of active TB cases in all settings in high-income countries [17], the transmission risk of TB infection in aircraft, at 0.1–1.3% [18], is substantially lower due to the use of “High Efficiency Particulate Air” (HEPA) filters, which completely exchange air 15 to 30 times per hour [25].

Of the 192 international CT notifications, 164 (85.4%) were initiated by developed countries, potentially indicating the presence of more resources to perform CTs. Indeed, CT is not a prioritized area of TB prevention in many resource-limited settings and well-defined standards of CT procedures and clear definitions of roles and responsibilities are lacking [14]. We cannot exclude the fact that our investigation, similar to a number of previously published CT investigations, is biased towards data from high-resource countries. This means that such analyses often may not reflect the true picture of potential exposures to TB during international air travel. When assessed together with the overall low risk of transmission within aircraft for TB, the resource-intensive nature of international CT following long-distance air travel, and the many potential missed occurrences of travel of infectious active TB cases on flights (occurrences that are frequently never identified), the necessity and practicality of routine CT following air travel can be brought into question.

The need for better physician and patient awareness around public travel and infectious protection measures for TB is reinforced by the fact that, in our study, 114 of the 192 index cases were considered to have been infectious during contact. Of these, 12 cases were diagnosed with drug resistant TB, which further increases the risk of spread of drug resistant TB strains. Although international guidelines state that confirmed TB cases should completely avoid air travel or, if unavoidable, wait until at least 2 weeks of treatment has been received and evidence for clinical improvement can be identified [6, 27], our analysis revealed that 7 index cases, including 2 with MDR-TB, were diagnosed with TB prior to their long-distance flights. Such events once again underline the need for physicians to better inform TB patients to either avoid commercial travel, use alternative private transportation or, if no alternative is available, to have a safety protocol established by the national public health authority and the airline, in order to decrease transmission risk of TB [3, 27]. Such events also underline the need for patients to be better informed regarding the risk public travel poses for other individuals during the infectious disease period for TB.

Our analysis revealed 32 (4.2% of all contacts) children who were identified as contacts of index TB cases, the majority of whom (*N* = 18, 56.3%) were exposed during private contact. Children are a vulnerable group who display unspecific symptoms of TB disease, leading to a delayed diagnosis that often occurs when the disease had already progressed to serious stages, increasing the risk of TB-associated death in children [7, 29]. As a result, timely CT plays an important role in the prevention or detection of childhood TB and the reduction of TB-associated childhood deaths. In 2017, almost 50% of all child TB cases in Germany were found through active case-finding via CT, in contrast to 4.0% of all adult TB cases [7]. Despite this well-known impact of CT in the prevention of childhood TB, 5 children in our study could not be investigated due to lack of adequate contact information; 3 of these children had contact with a drug-resistant index case, which makes the inability to contact the children or their guardians even more serious.

A key finding of our study was the large difference in the completeness of data reported in the international CT notifications. Among the notifications analyzed in our study, 17.2% did not contain any information on microscopy smear testing for the index cases and 43.3% of all notifications were missing information on chest radiograph results. Moreover, in 38.0% of the notifications, no information on DST results was reported. All of these are key for assessing infectiousness and seriousness of TB disease, and, therefore, also important for assessing the need of CT. DST results are further important for the provision of preventive therapy or chemoprophylaxis, especially in children, should contacts who test positive for LTBI be identified during the CT procedures. When assessing the completeness of information according to RKI’s risk assessment algorithm, only 11.5% of the 130 air travel-related notifications included information on all RAGIDA criteria. For the DZK guidelines (non-flight exposure), only 16.1% had information on cumulative duration of exposure and either smear microscopy results or laboratory culture results for the index case. In addition to missing clinical information, some CT notifications contained insufficient or no information on the contact details or address of the identified contact persons.

As a result, international standards for the reporting of information in international CT notifications are necessary in order to streamline the process and reduce the additional time and resources required when working with CT notifications that contain insufficient information. The standards should include information on the important clinical characteristics of the index case, including laboratory diagnostics, DST results, and infectiousness and symptoms of the index case during the exposure period. They should further include any relevant information on the identified contact persons that is required to trace them and carry out the CT procedures, including full name, date of birth, passport number, residential address, email address, and telephone number. Lastly, the standards should contain information on the duration and intensity of exposure for each contact, together with any knowledge of vulnerability of contacts, i.e. children < 5 years or immunosuppressed contacts. Based upon these findings, we have developed two standardized templates for international CT notification following flight and non-flight exposure, which we encourage other health authorities to utilize for their own CT notifications; both templates can be found in the Supplementary Materials under Additional File 1 and Additional File 2.

More recently, exposure to an active TB case following long-distance railway or bus trips, i.e. long-distance ground travel, has received increasing attention [30–32]. Beginning in 2019, which is outside the study period, RKI started to receive CT notifications specific to such incidents, a number of which concern long-distance bus travel within the border-free region of the European Union. However, CT following ground travel is associated with a variety of logistic obstacles, making follow-up of contacts very difficult or even unfeasible, as detailed passenger data is often not collected or is very incomplete [24, 30, 33]. Nevertheless, the risk of transmission during long-distance bus travel is considered to be greater than that for air travel, mainly due to the lack of standard HEPA filtration in buses and trains [24, 30, 34]. Unfortunately, scientific literature on CT following railway or bus travel is limited, making the assessment of its corresponding impact on public health difficult. The lack of high-quality evidence, in combination with the substantial amount of time and human resources needed for CT following ground travel, suggests that the decision to initiate CT should be reserved for select situations only, after careful consideration of all relevant factors [24, 30, 33, 34]. Once again, this indicates the need for a rethinking of the current conventions around CT for TB following international travel, keeping in mind that public health resources for TB might be better spent in other, more effective areas of prevention [33].

Our study has a number of limitations. First, the present analysis is based on CT notifications received by the German national public health institute, the RKI. Some foreign health authorities may have sent international CT notifications directly to the responsible local health authorities in Germany, in which case the local health authorities are not required to forward this information to the RKI. As a result, the total number of CT notifications reported in our study, as well as their characteristics, may not represent the full situation in Germany. Nonetheless, in our experience, RKI does receive the large majority of TB CT notifications for Germany, especially for CT following air travel. Thus, the analysis presented in this study provides the most comprehensive and complete insight to date into the international TB-related CT notifications received in Germany. Second, although RKI informally requests the final results of the CT procedures from the local health authorities, for example information such as the number of contacts reached and tested, the number of contacts who tested positive for LTBI or active TB, and the number of contacts started on preventive therapy, no information on TB-related CT is notifiable under the current disease surveillance laws in Germany. Hence, RKI does not have any information on the outcomes of the 192 CT notifications forwarded to the local health authorities during the study period, and therefore, could not estimate the yield or effectiveness of CTs following international travel. However, this study presents a very thorough inventory of other relevant data, which form a crucial aspect of international CT, and presents practical and important conclusions to improve international CT procedures, such as standardized notification templates. Lastly, our analysis was limited by the incomplete data available on the demographic and clinical characteristics of the index cases, which meant that more extensive analyses of these data other than descriptive analyses were not meaningful due to the small number of cases with available information. The lack of demographic characteristics of index cases is likely due to the minor role this information plays in the assessment of the need for CT; however, the lack of other important information, such as clinical characteristics, is in itself an important finding of this study, highlighting once again the importance of international standards for the reporting of CT information for TB.

## Conclusions

In conclusion, CT is an important cornerstone in prevention of TB, especially among high-risk vulnerable population groups, such as children. However, the current procedures regarding CT following international travel are resource-intensive and their public health impact is not backed by strong evidence. The results of this study, which, to our knowledge, is the first of its kind to comprehensively investigate the characteristics of international CT notification for TB, add to the body of literature from around the world in questioning the efficiency and practicality of systematic TB-related CT following international travel, except in extraordinary circumstances. This is particularly true for long-distance air travel, where the described risk of transmission is very low. We instead propose that a central part of a prevention strategy should be raising awareness among physicians to diagnose TB early and encouraging them to educate their TB patients, in particular those with drug-resistant TB, regarding the importance of avoiding public travel during the infectious phase of the disease. Furthermore, the current communication, decision-making, and evidence generation regarding international CT is hampered by a lack of consistency when it comes to the information exchanged between international health authorities. Standardization of the TB-related CT notifications may thereby contribute to a more efficient and practical communication process.

## Supplementary information

**Additional file 1.** International contact-tracing notification form for tuberculosis during air travel. Template for the notification of international contact-tracing for tuberculosis during air travel developed by the Tuberculosis Team at RKI.

**Additional file 2.** International contact-tracing notification form for tuberculosis during non-flight related exposure. Template for the notification of international contact-tracing for tuberculosis during non-air travel developed by the Tuberculosis Team at RKI.

## Data Availability

The Data Protection Office of the Robert Koch Institute restricts sharing of any case-based RKI data externally. However, aggregated data can be requested from the authors or the Research Data Management Unit of the Robert Koch Institute (MF4@rki.de) under reasonable conditions.

## Abbreviations

CT: Contact-tracing
DS: Drug susceptibility testing
DZK: German Central Committee against Tuberculosis
ECDC: European Center for Disease Prevention and Control
EWRS: Early Warning and Response System
H: Isoniazid
HEPA: High efficiency particulate air
IHR: International Health Regulations
LTBI: Latent tuberculosis infection
MDR-TB: Multidrug-resistant tuberculosis
PCR: Polymerase chain reaction
R: Rifampicin
RAGIDA: Risk assessment guidelines for infectious diseases transmitted on aircraft
RKI: Robert Koch Institute
S: Streptomycin
TB: Tuberculosis
XDR-TB: Extensively drug-resistant tuberculosis
