# Supplementary material for: International tuberculosis contact-tracing notifications in Germany: analysis of national data from 2010 to 2018 and implications for efficiency
Source: BMC Infect Dis. 2020 Apr 6;20:267. doi: 10.1186/s12879-020-04982-z (PMC7137477; doi:10.1186/s12879-020-04982-z)
Supplement: Supplementary file 2 — Additional file 2. International contact-tracing notification form for tuberculosis during non-flight related exposure. Template for the notification of international contact-tracing for tuberculosis during non-air travel developed by the Tuberculosis Team at RKI. [file 12879_2020_4982_MOESM2_ESM.pdf]

International contact-tracing notification form for tuberculosis during non-flight related exposure

Sending authority (institution, contact person, email, telephone):	Date: (dd/mm/yyyy)<br>Reference ID:

1. Information regarding index patient

Last name:	First name:	Date of birth: (dd/mm/yyyy)
Sex: ☐ female ☐ male ☐ other		Nationality:
Diagnosis:	Date of diagnosis: (dd/mm/yyyy)	Species:

	pos	neg	pending	not tested	Date of test (dd/mm/yyyy)
Microscopy:	☐	☐	☐	☐	
Culture:	☐	☐	☐	☐	
PCR:	☐	☐	☐	☐	
Chest X-ray:	☐	☐	☐	☐	

Drug susceptibility and resistance information:

	H	R	E	Z	S
susceptible	☐	☐	☐	☐	☐
resistant	☐	☐	☐	☐	☐
pending	☐	☐	☐	☐	☐
unknown	☐	☐	☐	☐	☐

Further DST results:

Additional lab information:

Symptoms:

Infectiousness: ☐ yes ☐ no

Date of onset: (dd/mm/yyyy)

Further remarks:

Evidence of transmission to close contacts: ☐ yes, active TB ☐ yes, LTBI ☐ no ☐ unknown

2. Information regarding identified contact(s)

+ click to add a contact

Type of exposure (household, school, work):	Duration of exposure:<br>Date of last contact: (dd/mm/yyyy)

Last name:	First name:
Date of birth: (dd/mm/yyyy)	Child <5 years: ☐
Nationality:	Passport No.:
Address:	Telephone No.:<br>Email:

Further remarks:

3. For further inquiries please contact:

☐ sending authortiy ☐ other:

☐ *Please inform us of the results of the contact investigation*
